# New Appliances and Things Medical

**Published:** 1902-01-11

**Authors:** 


### NEW APPLIANCES AND THINGS MEDICAL.

### OXO.

(Liebig's Extract of Meat Company, Fenchurch Avenue, London, E.C.)

Oxo is certainly one of the most satisfactory of the fluid beef preparations on the market. We have before in these columns referred to the agreeable flavour which is possessed by this speciality of the Liebig Company, and again we would bear testimony to its excellent nutritive properties and its suitability for invalid use. Owing to economical use and comparative cheapness, it is especially to be recommended to the notice of district nurses and charitable people who will be glad to know of a reliable preparation of this kind for distribution among the poor.

### L'OZONATEUR.

(Langton, Ford and Burden, 12 Little Britain, London, E.C.)

This little apparatus is an automatic disinfector for use in sick rooms, lavatories, schools, factories, etc. The method of use is perfectly simple; the liquid disinfectant, ozonatine, is poured into the apparatus through an opening at the top. The liquid passes into a glass receiver below, into which a large cylindrical wick dips; the latter extends into a chamber above which is formed of perforated tin. The ozonatine spreading up the wick by capillary attraction, evaporates in the upper chamber and diffuses through the perforated openings into the surrounding air. By this means a constant supply of the vapour passes into the room in which the disinfector is hung, and continues so to do, as long as there is any fluid left in the receiver. The apparatus is simple, practical, and economical to use.

### HORBY AND McKAY'S BYE-PASS AND REGULATING VALVE.

(London Agent: T. A. Stevenson, 24 Victoria Street, London, S.W.)

This ingenious invention is applicable for the regulation of the flow of steam, gas, air or water. To our mind it is a very practical form of valve, being strongly made, and tested up to 200-lbs. pressure to the square inch. It can be adjusted to a nicety, so that the bye-pass permits of the flow of any desired quantity of gas or fluid, and at the same time the main valve can be opened or closed with confidence that the bye-pass flow will return to the pre-determined point immediately the main valve is closed. There are many obvious domestic uses to which this mechanism can be applied, as, for instance, in connection with hot-water services, automatic flushing tanks, gas services, etc.; it might also have considerable value in medical practice if applied to gas cylinders containing oxygen under pressure. Under the ordinary arrangements it is very difficult to maintain a constant and equivable supply of oxygen from such cylinders to sick persons. One of these valves tested for a sufficiently high pressure might allow a constant and regulated stream of the gas to pass through the bye-pass and supply the patient without waste.

